# Plain metallic biomaterials: opportunities and challenges

**DOI:** 10.1093/rb/rbac093

**Published:** 2022-11-15

**Authors:** Jiazhen Zhang, Bao Zhai, Jintao Gao, Zheng Li, Yufeng Zheng, Minglong Ma, Yongjun Li, Kui Zhang, Yajuan Guo, Xinli Shi, Bin Liu, Guobiao Gao, Lei Sun

**Affiliations:** Center for Medical Device Evaluation, National Medical Product Administration, Beijing 100081, China; Center for Medical Device Evaluation, National Medical Product Administration, Beijing 100081, China; Guangdong-Hong Kong-Macao Greater Bay Area, Center for Medical Device Evaluation and Inspection of NMPA, Shenzhen 518045, China; Center for Medical Device Evaluation, National Medical Product Administration, Beijing 100081, China; School of Materials Science and Engineering, Peking University, Beijing 100871, China; State Key Laboratory of Nonferrous Metals and Process, GRINM Group Corporation Limited (General Research Institute for Nonferrous Metals), Beijing 100088, China; State Key Laboratory of Nonferrous Metals and Process, GRINM Group Corporation Limited (General Research Institute for Nonferrous Metals), Beijing 100088, China; State Key Laboratory of Nonferrous Metals and Process, GRINM Group Corporation Limited (General Research Institute for Nonferrous Metals), Beijing 100088, China; Center for Medical Device Evaluation, National Medical Product Administration, Beijing 100081, China; Center for Medical Device Evaluation, National Medical Product Administration, Beijing 100081, China; Guangdong-Hong Kong-Macao Greater Bay Area, Center for Medical Device Evaluation and Inspection of NMPA, Shenzhen 518045, China; Center for Medical Device Evaluation, National Medical Product Administration, Beijing 100081, China

**Keywords:** plainification, metallic biomaterials, regulatory science, medical devices, pure titanium, high-purity magnesium

## Abstract

The ‘plainification of materials’ has been conceptualized to promote the sustainable development of materials. This perspective, for the first time in the field of biomaterials, proposes and defines ‘plain metallic biomaterials (PMBs)’ with demonstrated research and application case studies of pure titanium with high strength and toughness, and biodegradable, fine-grained and high-purity magnesium. Then, after discussing the features, benefits and opportunities of PMBs, the challenges are analyzed from both technical and regulatory aspects. Regulatory perspectives on PMB-based medical devices are also provided for the benefit of future research, development and commercialization.

## Graphical abstract

**Figure:**
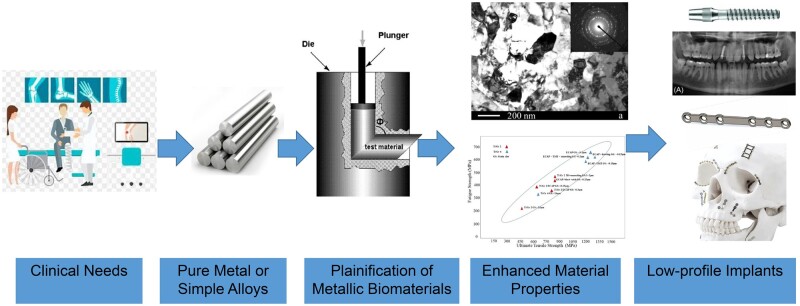

## Introduction

The field of traditional metallic materials is facing significant changes. With the continuous improvement of the alloying degree of metals, the performance improvement of engineering metallic materials by alloying approaches has reached the limit, and alloying also increases the resource dependence, cost and difficulty of recycling. In order to maintain the sustainability of material development, Lu *et al*. [1] have proposed the concept of plain materials as follows: ‘a sustainable “plain” approach to advancing materials without changing chemical compositions, i.e. architecturing imperfections across different length-scales’. Novel properties and performance can be achieved in the ‘plain’ materials with less alloying or even non-alloying [2, 3].

Traditional metallic materials can be controlled in the microstructure through reasonable composition design and appropriate processing technology to obtain better performance. Plainification of metals (PM) is to apply a simpler composition design, such as pure metal or simple alloy components, and scientifically match processing technology to achieve precisely controllable cross-scale material microstructure and defects (e.g. vacancies, dislocations and defects, grain or phase boundaries) as well as desired performance. In recent years, significant progress has been made in precisely controlling the interface or defects of different scales, making it possible to effectively improve the comprehensive properties and service stability of metallic materials through PM [4].

PM relies on processing technologies such as equal-channel angular pressing (ECAP), high-pressure torsion (HPT) and hydrostatic extrusion, which are characterized by severe plastic deformation (SPD) for refined grains. When the grain size is reduced to the sub-micron or nanoscale, the yield strength of the material is significantly improved while the plasticity is maintained [5]. Recently, the nanotwinned Ti material (nanotwinned Ti) [6] prepared by low-temperature multi-directional forging (cryoforge) was reported to show ultrahigh strength and ductility, promising expanded application in wide-ranging areas.

Lately, PM has been applied to biomaterials for implantable and interventional medical devices with notable success. Zeng *et al*. [7] also pointed out the benefits of the plain biomedical magnesium materials for their potential clinical applications. This perspective will firstly introduce the application of metallic biomaterials (MBs) and their medical devices, and then propose the concept and definition of plain metallic biomaterials (PMBs) along with their potential applications in medical devices. Next, opportunities and challenges for PMBs are analyzed. Finally, regulatory science programs related to PMB-based medical devices are discussed.

## PMBs and their applications

**Figure 1.**
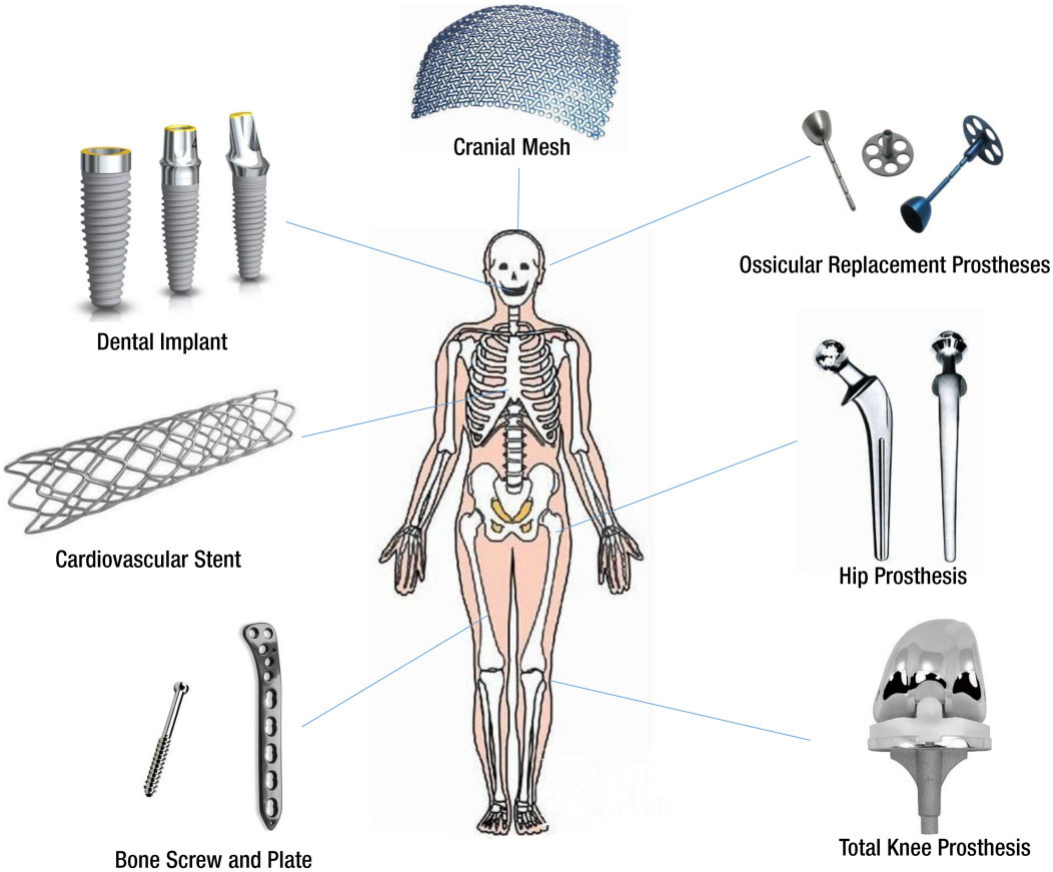
Applications of metallic biomaterials in cranial meshes, ossicular replacement prostheses, cardiovascular stents, bone screws and plates, dental implants and hip and knee prostheses.

As one of the important areas in the field of biomaterials, MBs have been widely used in medical devices due to their excellent mechanical strength, plasticity and biocompatibility [8]. MBs include stainless steel, cobalt-based alloys, titanium and titanium alloys and zirconium and alloys, to name a few, which are applied widely in the fields of orthopedics, dentistry and cardiovascular interventions [9], as shown in Fig. 1.

Recently, the safety concerns of long-term metal implants have attracted regulatory attentions. In 2019, Food and Drug Administration (FDA) released a white paper on biological responses to metal implants, addressing the potential toxicity and immunological reactions caused by metal implants used in clinical applications [10]. Metal implants may release ions or particles that contain matrix constituent elements such as aluminum, nickel, cobalt and chromium during service, which may induce adverse tissue reactions and cause health hazards after accumulation to threshold dosage [11]. Given the existing problems of implantable metallic devices, the research and development of a new generation of MBs and their medical products have been suggested.

The widely used MBs, such as stainless steel, cobalt-based alloys, titanium and titanium alloys, were first applied in the engineering field before being gradually expanded to the medical field. Some elements added to pure metals in the form of alloying are potentially harmful to the human body. At first, biocompatibility as a key property of biomaterials was not given enough attention, because the focus then was on the mechanical and tribological properties and corrosion resistance, which were the big historical issues in the development of MBs. With the emergence of PM processes and technologies, the preparation of biomedical plain metals or alloys with simple compositions has exhibited promising applications.

### Case study 1: pure titanium with high strength and toughness

Pure titanium has fine biocompatibility and biomechanical adaptability, which makes it successful candidate in dental and orthopedic implants [12]. However, the strength of commercial pure titanium is low. In clinical applications that require high strength, it is still necessary to use stainless steel, cobalt–chromium–molybdenum alloy or titanium alloy, which generates the possibility of adverse tissue reactions during long-term implantation in the human body.

Contemporary researchers have attempted to take advantage of the excellent biocompatibility of pure titanium while improving its comprehensive mechanical properties and wear resistance, so as to expand its potential applications [13]. Pure titanium with high-strength and toughness reinforced by the SPD processing technology shows excellent comprehensive properties. The microstructure of the pure titanium processed by ECAP is refined from coarse grains to ultrafine-grained titanium (UFG-Ti) [14]. Self-passivation and corrosion resistance are both significantly improved compared to those of commercially pure titanium (CP-Ti) [15] Additionally, the mechanical properties of pure titanium processed by ECAP such as tensile strength, bending strength, hardness and fracture toughness have also been significantly enhanced [16]. Combined with appropriate annealing processes, the notch sensitivity and impact strength of UFG-Ti can also be improved [17]. The mechanical properties and endurance limits of UFG-Ti and conventional pure titanium are shown in Table 1. With the grain refinement of pure titanium, the mechanical strength and fatigue properties of pure titanium materials are significantly improved. The fatigue strength of UFG Ti is comparable to that of titanium alloys. Furthermore, UFG-Ti has an excellent combination of strength and ductility with the ultimate tensile strength of 1290 MPa and the tensile elongation of 13% [18]. Previous study has found that CP-Ti rods treated by both rotary swaging (RS) and annealing achieved a strong <10-10> fiber texture, a microstructure characteristics of UFG, while obtaining excellent mechanical properties including tensile strength of 870 MPa, elongation of 8.5%, and high fatigue limit of 490 MPa, which are very close to those of the Ti-6Al-4V alloy [19]. Similarly, UFG Ti subjected to the HPT process also showed good wear resistance [20]. A previous study also found that compared to CP-Ti, pure titanium with nanosurface structure can enhance protein adsorption and cell adhesion, promote the expression of osteogenic-related genes, and improve biocompatibility and osseointegration [21].

**Table 1.** Mechanical properties and fatigue strength of UFG-Ti compared with conventional Ti

Material	Ultimate tensile strength (MPa)	Yield strength (MPa)	Elongation (%)	Fatigue strength 106 cycles (MPa)	Grain size (μm)	Process	Ref.
Ti Grade 2	460	380	26	238	∼25	Conventional Ti	[22]
	710	625	14	404	∼0.25	ECAP	[22]
	810	650	15	380	∼0.3	ECAP	[23]
	870	740	8.5	490	1∼2	RS + annealing	[19]
	850	750	18	430	∼0.25	ECAP + sand blasting + acid-etching	[24]
Ti Grade 4	700	530	25	340	∼27	Conventional Ti	[25]
	1260	1120	13.5	625	∼0.1	ECAP	[26]
	1250	1000	13	620	∼0.2	ECAP + TMT + annealing	[27]
	1240	1200	12	620	∼0.15	ECAP + TMT	[28]
	1290	1210	13	610	∼0.17	ECAP + drawing	[18]
	1330	1267	11	620	∼0.15	ECAP + drawing	[25]

ECAP, equal-channel angular pressing; RS, rotary swaging; TMT, thermo-mechanical treatment.

**Figure 2.**
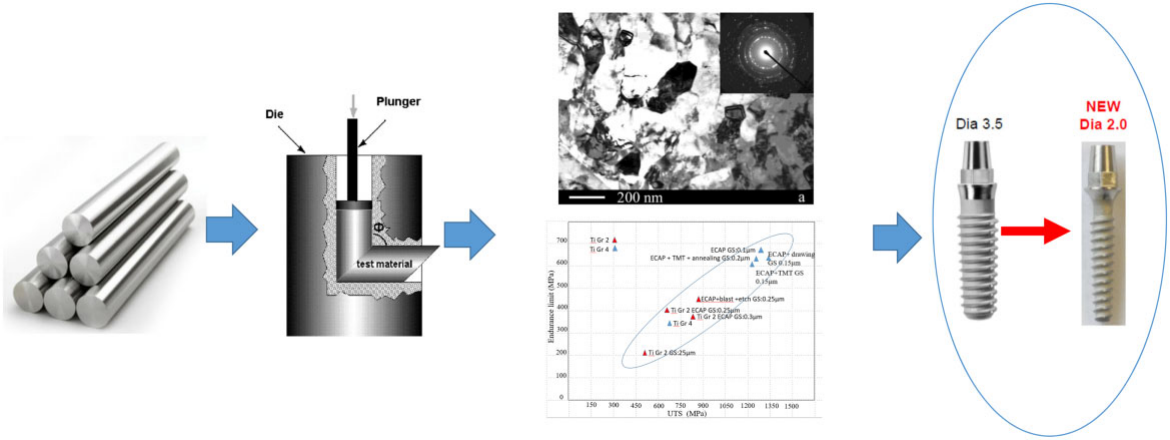
Schematic diagram of the plain titanium-based dental implants [32, 33], reprinted with the permission from Elsevier.

Pure titanium with ultrafine grain characteristics has many advantages. It can effectively avoid the ion toxicity of alloying elements while keeping the composition simple. Meanwhile, the improvement of its mechanical properties can reduce the size and footprint of the implant. Products currently developed using such material are hopeful and expanding. For example, dental implants made of high-strength pure titanium with nanostructures can be as thin as 2.0 mm in diameter, which can effectively retain the alveolar bone and maintain good osseointegration [29]. Figure 2 shows the schematic diagram of the plain titanium-based dental implants. In addition, high-strength ultrafine-grained pure titanium has been used in small bone plates [30]. The bone-plate implant can be made thinner with less occupation of tissue space for a low-profile design. Recently, researchers have used additive manufacturing technology to prepare pure titanium, achieving lower cost, higher performance and better biocompatibility. This has opened up an optimistic field for PMB research, with a potential to replace titanium alloys [31].

### Case study 2: biodegradable, fine-crystalline and high-purity magnesium

Biodegradable magnesium is a recent research hotspot, magnesium is an element in the human body that exists in bones and muscles [34, 35]. Magnesium implants have fine biocompatibility and degradability, which require no secondary procedures for removal. Current research topics include the development of new magnesium and magnesium alloys with good corrosion resistance, improving the mechanical properties of pure magnesium through material processing technology, and reducing the corrosion of magnesium alloys through coating technology [36, 37]. The corrosion of magnesium and magnesium alloys is affected by many factors, such as: composition, structures including grain size, second phase, texture, dislocation and twin, processing technologies such as extrusion, rolling, casting, forging and heat treatment, chemical, mechanical and biological environment [7]. In order to improve the mechanical properties of magnesium and its alloys, one of the solutions is to add alloying elements such as Zn, Ca, Sr, Li and Nd [38], which can improve the effect of both solid solution and precipitation strengthening, but most of as-generated intermetallic compounds as the second phase will increase corrosion, due to the potential difference between the second phase and the α-Mg matrix. As a result, pitting corrosion, filament corrosion and exfoliation corrosion could accelerate the degradation of the alloy [39, 40].

Compared with magnesium alloys, high-purity magnesium has no alloying elements added, which means no secondary corrosion in the matrix to cause micro-galvanic corrosion [41]. The degradation rate of high-purity magnesium is relatively slow [42]. The Mg^2+^ ions generated during degradation can promote the osteogenic differentiation of stem cells in the periosteum at the fracture site, which benefits the regeneration of bone tissue around the implant and accelerates fracture healing. As a result, high-purity magnesium has a great potential for orthopedic internal fixation implants [43–45]. For example, a high-purity magnesium screw currently under development presents a marvelous fixation effect when used in the surgical treatment of the Association Research Circulation Osseuse (ARCO) stage II/III patients with femoral head necrosis [46, 47]. Promising *in vivo* study results show that high-purity magnesium screws will soon be developed for the treatment of femoral neck fractures [48] and for anterior cruciate ligament reconstruction [49].

However, the mechanical properties of pure magnesium itself are relatively low. In order to achieve stable fixation, the size of pure magnesium implants is larger than that of conventional titanium implants, thus releasing an increased volume of hydrogen. The gas generated by its rapid degradation may cause complications such as superficial skin necrosis and long-term osteolytic lesions, which need to be reduced in order to achieve successful clinical outcomes [50]. As a result, researchers have explored the use of different processing technologies or combinations to improve the mechanical properties of pure magnesium and reduce its degradation rate. For example, ECAP, HPT, rotary forged and other approaches have been used to refine the grain of pure magnesium, and investigate the strengthening mechanism and deformation behavior [51, 52]. Grain refinement of magnesium can activate more slip systems during deformation, which increases the material strength and ductility, and reduces the corrosion rate of pure magnesium [53]. Previous studies also found that through the low-temperature extrusion combined with the cold-rolled process, the pure magnesium could achieve super-formable characteristics without adding alloying elements [54]. Fu *et al*. [55] found that due to the strengthening effect brought by grain refinement, the mechanical properties and corrosion resistance of high-purity magnesium were also significantly improved, with the strength reaching 210 MPa, which is comparable to some magnesium alloys, and the corrosion resistance was better than that of extruded high-purity magnesium. This suggests great potentials for product design and performance improvement of pure magnesium. Meanwhile, new evaluation methods are constantly improving. For example, new testing technologies such as thin electrolyte layer model, to simulate the *in vivo* corrosion of pure magnesium and other degradable materials have been developed, which could provide a convenient and efficient method for future product development [56].

In addition, Zn metal and alloys, given their degradability, biocompatibility and moderate mechanical properties, have become one of the very promising candidates for biodegradable implants [57]. The Zn element has both pro-osteogenic and antibacterial effects and is expected to be developed into cardiovascular, orthopedic and dental implants [58, 59]. It was found that a simple zinc alloy (Zn-0.033 Mg, in wt.%) processed by ECAP, exhibiting both excellent mechanical properties and biodegradation for potential implant applications. Due to the combined effect of grain refinement, texture and dislocation strengthening, the alloy obtained good mechanical properties, with yield strength of 250 ± 22 MPa, elongation of 25.37 ± 0.79% and low corrosion rate of 0.004–0.01 mm/year [60]. Thus, the research on plain Zn metal is increasingly active [61–63].

At the same time, with the application of novel processing technologies including ECAP, RS and thermo-mechanical treatment in MBs such as titanium, magnesium and zinc, and the research on the deformation mechanism, enhancement of mechanical property and improvement of corrosion resistance of pure metals or simple alloys, the effects of grain refinement, texture and wide stacking faults on materials’ performance have been thoroughly investigated and gradually revealed [55, 60]. In addition, the application of RS may overcome the limitation of ECAP and HPT regarding the small size (especially length) of as-prepared materials, which could provide new opportunities for the promotion and application of PMBs [19].

Taken together, we propose the definition of PMBs as pure metals or alloys with relatively simple compositions, which have unique characteristics including precise control of the microstructure through processing technologies, excellent mechanical properties and biocompatibility, and safe for long-term medical services.

## Opportunities and challenges of PMBs

### Features, benefits and opportunities

We have established that PM a significant has positive impact for MBs and their medical devices. Multiple improvements can be made in terms of the composition, mechanical performance, ease of processing, potential of application, impact on health and environment, and cost-effectiveness. The features, benefits and potential opportunities of PMBs are summarized in Table 2.

**Table 2.** Features, benefits and potential opportunities of PMBs

Key elements	Features	Benefits	Opportunities
1 Composition	The composition is simple: PMBs do not add or only add a small amount of alloying elements.	PMBs can reduce the potential biological risks caused by unintended ion precipitation or particle shedding such as corrosion and wear during the service of the implants, and thus have better biocompatibility.	Medical devices can be designed with lower biological risks.
2 Mechanical properties	The mechanical properties of PMBs can be optimized by controlling the microstructure and defects of different scales.	Mechanical properties of PMBs are excellent, and the size of the implants can be reduced.	Implants and intervention devices with optimal structures can be designed.
3 Processing ability	PMBs can be prepared by processing technologies such as severe plastic deformation (SPD), and the overall performance of PMBs is stable.	Conventional machining and laser engraving of implants and interventional devices can be applied.	Easy to promote and replace existing materials.
4 Application scenarios	In the human body 37°C environment, far lower than the high-temperature environment for the applications of engineering materials.	PMBs have good stability and can maintain the microstructure, mechanical properties and biocompatibility after plainification.	Medical device-related applications have broad prospects.
5 Health and environment	No added elements such as aluminum, vanadium, nickel and chromium.	Avoids exposure of toxic elements in the environment, reduces health and environmental pollution risks, and issues of recycling [64]	Contribute to the sustainable development of human beings, the environment and society.
6 Cost	Fewer (no) alloying elements and alloying processes.	Avoid the high cost of some special alloying elements, alloying processes and complex hot and cold working processes to ensure alloying composition and structure.	Cost saving.

### Technical challenges

Although PMBs have outstanding application prospects, they face challenges in the following aspects. The preparation process of new materials needs to be strengthened in terms of stability. Most of the PMBs possess the characteristics of new materials and new processes. The processing methods and follow-up treatments of PMBs are mostly customized. There is a lack data and experience in the structure–property relationships of PMBs. Due to the uniqueness of processing methods such as SPD, there may be uneven structures, high residual stress and obvious anisotropy in different processes. Subsequently, the manufacturing of medical devices is troubled with increased uncertainty and instability. Therefore, it is necessary to continuously explore reasonable methods and technologies to improve the consistency of material properties for the superior performance of medical devices.

Standards and evaluation methods for PMBs need to be established urgently. When metals transform from coarse grains to ultrafine grains and nanocrystals, the original method of microstructure evaluation using optical metallographic microscopy is no longer applicable. Novel methods and evaluation standards are required, such as using electron backscatter diffraction for microstructure evaluation [65]. In addition, some texture-strengthened materials need better heat treatment for texture adjustment and performance optimization. The effect of microstructure on biocompatibility of PMBs is more significant than that on the grain size [66]. A validated annealing process can improve the mechanical properties and corrosion resistance of ultrafine-grained PMBs. Otherwise, it may damage-related material properties [67]. There are standards for pure titanium and titanium alloys, such as ISO5832-2 and ISO5832-3. However, these standards do not apply to microstructures. Therefore, it is necessary to carry out methodology-related basic research on standards, methods and criteria.

The challenges brought about by product design merit attention. The vast majority of implants currently on the market are designed based on the mechanical properties of traditional materials. With novel materials such as PMBs, product designs must be adapted. For example, in order to achieve a low-profile design, thinning or reducing the diameter of the implant is needed. Taking the bone plate for the treatment of long bone fractures of the extremities as an example, the use of high-performance pure titanium can meet the needs of tensile stress, but the elastic modulus of the material does not change much after plainification. Thinning of implants will affect the stability of the fracture after fixation, and increase the displacement and dislocation of the fracture, resulting unsatisfactory clinical outcomes. Therefore, the application of each new material should be holistically considered and reviewed in multiple dimensions such as material properties, biomechanics, and clinical applications, rather than simply emphasizing the improvement of performance in one aspect.

### Regulatory challenges and regulatory science

In addition to technical challenges, innovative materials, processes, designs and products will pose new regulatory challenges. These pose the challenges of safety and efficacy evaluation and supervision. To enter innovative materials and their medical products into the market, the product development phases such as design verification and validation, production, quality inspection, clinical evaluation, registration and marketing are proven crucial, beyond the basic research in materials science, toxicology, biology and *in vivo* studies [68–70]. The product development cycle is often prolonged due to the lack of evaluation methods, standards and guidance documents for innovative materials and their medical products. In addition, in the initial stage of the introduction of new materials to the market, the demand for them is relatively small, the investment in equipment and Research and Development (R&D) costs are high, and the investment period is long, which also hinders the application of new PMBs and the launch of their new products.

Regulatory science is ‘the science of developing new tools, standards, and approaches to assess the safety, efficacy, quality, and performance of regulated medical products’ [71]. To promote innovative biomaterials and their medical products, fully identify relevant risks, ensure the safety and effectiveness of products, and promote the development and clinical application of innovative biomaterials, regulators in the USA, China, European Union (EU) and Japan all have carried out regulatory science programs and research. Among them, China’s National Medical Products Administration (NMPA) has implemented its regulatory science action plan (RSAP). The RSAP, released and implemented in 2022, includes guidance documents for new materials and products such as the one with the concept of PMBs on high-strength and toughness pure titanium. Additionally, standards on high-toughness pure titanium are being formulated. The development of regulatory science for medical devices will play an important role in promoting the product development, marketing and supervision of PMBs and their products [72].

In March 2021, NMPA also issued the ‘Announcement on the Registration Items of Medical Device Master File’ (No. 36 of 2021) [73]. Medical device master files are designed to respect intellectual properties and protect trade secrets. In the case of multiple citing of the same raw material master file, the application of master file could avoid repeated tests on the same material by different device manufacturers. This new regulation will save reviewer resources, improve the efficiency of review and registration, and accelerate the marketing and applications of new materials [74]. Medical device master files could create new opportunities for novel materials such as PMBs in the field of medical devices.

In April 2021, the Center for Medical Device Evaluation of NMPA created a biomaterial research innovation and cooperation platform [75]. This platform aims to promote collaboration among academia, research institutes, industry, healthcare institutions and government regulatory agencies in the research, development, commercialization and regulation of innovative materials and medical devices. Novel biomaterials as PMBs could be efficiently translated to medical devices via the platform.

## Conclusions

PMBs can significantly improve the comprehensive properties of MBs. While maintaining the simple composition, PMBs have excellent biocompatibility and biosafety that traditional materials do not provide. As a result, new opportunities are created for PMBs in the medical devices market. We have discussed successful case studies where pure titanium can achieve the strength of titanium alloys, and where pure magnesium can improve mechanical properties while reducing corrosion rate. Such approaches provide possibilities for improved design and clinical applications of innovative medical devices. Currently, PMBs are being tested in the fields of oral, orthopedics and cardiovascular implants, and the emergence of these innovative PMB-based medical devices presents regulatory challenges. The development of regulatory science for medical devices could provide scientific evidence to ensure the safety, efficacy, performance and quality of PMB-based medical devices under the premise of both improved benefits and reduced risks.

## Supplementary Material

rbac093_Supplementary_Data
