# Supplementary material for: Plain metallic biomaterials: opportunities and challenges
Source: Regen Biomater. 2022 Nov 15;10:rbac093. doi: 10.1093/rb/rbac093 (PMC9847527; doi:10.1093/rb/rbac093)
Supplement: rbac093_Supplementary_Data [file rbac093_supplementary_data.docx]

November 1^st^, 2022

Dear Editor,

Thank you very much for organizing a very thorough and constructive review of our manuscript entitled “Plain Metallic Biomaterials：Opportunities and Challenges”, Manuscript ID RB-2022-183. Enclosed please find our revised manuscript that has addressed all of the reviewers’ comments. In addition, the following is a point-by-point response to the reviewers’ comments. **Each comment is bolded** and, *followed by our response in italic*. In the revised manuscript, the revisions are highlighted in light gray to facilitate the review.

Please let us know if you have any further questions. Thank you again for your consideration.

Sincerely,

Jiazhen Zhang

Center for Medical Device Evaluation,

National Medical Product Administration, Beijing, 100081, China

Email: zhangjz@cmde.org.cn	Phone: +86-01086452803

**Reviewer #1:**

**This manuscript, RB-2022-183, as an enlightening perspective, presents the concept, applications, challenges and opportunities of "plain metallic biomaterials (PMBs)". The reviewer recommends a minor revision because of the following reasons:**

**Question 1: In section 3.3, the last sentence of the first paragraph should be the first sentence of the second paragraph; authors should also correlate the content of both the third and fourth paragraphs to the regulatory perspectives of PMBs; furthermore, new references should be added to the regulatory science programs and achievements in the second paragraph.**

*The referred content in Section 3.3. was revised according to the reviewer’s recommendations. Specifically, the last sentence of the first paragraph in previous draft is now the first sentence of the second paragraph in the revised manuscript. Furthermore, the following sentences were added to the third and fourth paragraph of the revised manuscript, respectively: “Medical device master files could create new opportunities for novel biomaterials such as PMBs in the field of medical devices.”, and “Novel biomaterials as PMBs could be efficiently translated to medical devices via the platform”. In addition, reference 72 was also added.*

**Question 2: Please identify the reference(s), if any in the caption of Figure 2.**

*Figure 2 was revised and references 32 and 33 were also cited with the permission from Elsevier. Please see the following info.*

Figure2. Schematic diagram of the plain titanium-based dental implants[32,33], reprinted with the permission from Elsevier.

32.	Zhernakov VS, Latysh VV, Stolyarov VV *et al.* The developing of nanostructured spd ti for structural use. *Scripta Mater* 2001;44:1771-1774.

33.	Mishnaevsky L, Levashov E, Valiev RZ *et al.* Nanostructured titanium-based materials for medical implants: Modeling and development. *Mat Sci Eng R* 2014;81:1-19.

**Question 3:There are English typos and mistakes throughout the manuscript. For examples, “Equal-Channel Angular Pressing (ECAP)” should be “equal-channel angular pressing (ECAP)”; “titanium” should be deleted from the caption of Figure 1; “[12]…, and Pure titanium with high-strength and toughness…” should be “Pure titanium with high-strength and toughness…”.**

*English was polished throughout the manuscript and the mistakes referred by the reviewer were also corrected. Please see the following Figure 1 and revised manuscript for details.*

Figure 1. Applications of metallic biomaterials in cranial meshes, ossicular replacement prostheses, cardiovascular stents, bone screws and plates, dental implants, and hip and knee prostheses.

**Reviewer #2:**

**The paper" Plain Metallic Biomaterials：Opportunities and Challenges " proposed the concept and definition of plain metallic biomaterials (PMBs). The view is very innovative and enlightening. Pretty sure that this idea is bound to become one of the future development directions of metallic biomaterials. The resulting manuscript is more satisfying.**

*Authors thank Reviewer #2 for the very positive and encouraging comments.*

**Reviewer #3:**

**The manuscript involves the new concept for “Plain Metallic Biomaterials ：**

**Opportunities and Challenges”. This is a very interesting topic. The review article could be considered for publication in the journal after a major revision.**

**Question 1: Introduction. “This perspective will FIRSTLY introduce the application of metallic biomaterials and their medical devices, and then propose the concept and definition of plain metallic biomaterials (PMBs) along with their potential applications in medical devices.” However, it should be noted that plainification of biomedical Mg alloys has been previously pointed out by Prof. Zeng in Acta Metallurgica Sinica [Ref.1] in 2018. A comment should be made in Introduction.**

*“Firstly” in the context means the structure of this perspective, as shown below.*

This perspective will firstly introduce the application of metallic biomaterials and their medical devices, and then propose the concept and definition of plain metallic biomaterials (PMBs) along with their potential applications in medical devices. Next, opportunities and challenges for PMBs are analyzed. Finally, regulatory science programs related to PMB-based medical devices are discussed.

*Authors also recognize Professor Zeng’s work by adding the following sentence and reference 7 in the Introduction.*

“Zeng et al. also pointed out the benefits of the plain biomedical magnesium materials for their potential clinical applications”[7]

7. Zeng R, Cui L-Y, Ke W. Biomedical Magnesium Alloys: Composition, Microstructure and Corrosion. *Acta Metall Sin* 2018;54:1215-1235.In Chinese.

**Question 2: Figure 2 shows the schematic diagram of the plain titanium-based dental implants. However, the origin of the figure should be cited and allowed. And the samples #1-3, N and ϭ should be designated. What is the sample of the microstructure?**

**The relationship between the strength and number of cycles can be explained in detail in the context.**

*Figure 2 was revised and references 31 and 32 were also cited with the permission from Elsevier. Please note that the size of the product is public information as shown in its catalog at* [*http://www.timplant.cz/en/catalogue/system-nanoimplant/*](http://www.timplant.cz/en/catalogue/system-nanoimplant/)*. In addition, in the revised Figure 2, the microstructure graph is the TEM image of the nanostructure titanium. Furthermore, the relationship between the strength and the number of cycles is shown in the figure as well. Please see the following info, response to Question 3 as well revised manuscript for details.*

Figure2. Schematic diagram of the plain titanium-based dental implants[32,33], reprinted with the permission from Elsevier.

32.	Zhernakov VS, Latysh VV, Stolyarov VV *et al.* The developing of nanostructured spd ti for structural use. *Scripta Mater* 2001;44:1771-1774.

33.	Mishnaevsky L, Levashov E, Valiev RZ *et al.* Nanostructured titanium-based materials for medical implants: Modeling and development. *Mat Sci Eng R* 2014;81:1-19.

**Question 3: A comparison of the data can be made from the previous studies on Titanium, magnesium [refs 1-9], and zinc alloys in order to support the points of view.**

*After investigating references including those recommended by the reviewer, authors summarized the data of UFG-Ti and compared them with conventional Ti. Please see the following Table 1. Furthermore, authors also created the graph of ultimate tensile strength-fatigue strngth in Figure 2 based on the data collected in Table 1. Authors presented the related info in the second paragraph of Section 2.1.*

Table 1: Mechanical Properties and Endurance Limit of UFG-Ti compared with Conventional Ti.

| Material | Ultimate Tensile Strength (MPa) | Yield  Strength  (MPa) | Elonga-  tion(%) | Fatigue  Strength  10^6^ cycles (MPa) | Grain Size  (μm) | Process | Ref. |
| --- | --- | --- | --- | --- | --- | --- | --- |
| Ti  Grade 2 | 460 | 380 | 26 | 238 | ~25 | Conventional Ti | [22] |
|  | 710 | 625 | 14 | 404 | ~0.25 | ECAP | [22] |
|  | 810 | 650 | 15 | 380 | ~0.3 | ECAP | [23] |
|  | 870 | 740 | 8.5 | 490 | 1~2 | RS+  annealing | [19] |
|  | 850 | 750 | 18 | 430 | ~0.25 | ECAP+sand blasting+ acid-etching | [24] |
| Ti  Grade 4 | 700 | 530 | 25 | 340 | ~27 | Conventional Ti | [25] |
|  | 1260 | 1120 | 13.5 | 625 | ~0.1 | ECAP | [26] |
|  | 1250 | 1000 | 13 | 620 | ~0.2 | ECAP + TMT + annealing | [27] |
|  | 1240 | 1200 | 12 | 620 | ~0.15 | ECAP + TMT | [28] |
|  | 1290 | 1210 | 13 | 610 | ~0.17 | ECAP+ drawing | [18] |
|  | 1330 | 1267 | 11 | 620 | ~0.15 | ECAP+ drawing | [25] |

*Note:* *ECAP: equal-channel angular pressing, TMT: thermo-mechanical treatment, RS: rotary swaging.*

Figure2. Schematic diagram of the plain titanium-based dental implants[32,33], reprinted with the permission from Elsevier.

Previous study has found that CP-Ti rods treated by both rotary swaging and annealing achieved a strong < 10-10> fiber texture, a microstructure characteristics of UFG, while obtaining excellent mechanical properties including tensile strength of 870 MPa, elongation of 8.5%, and high fatigue limit of 490 MPa, which are very close to those of the Ti-6Al-4V alloy [19].

*Both Mg and Zn have limited data for comparison. As a result, authors presented their related info in the second and the third paragraph of Section 2.2 in the manuscript, respectively.*

Fu et al. found that due to the strengthening effect brought by grain refinement, the mechanical properties and corrosion resistance of high-purity magnesium were also significantly improved, with the strength reaching 210MPa, which is comparable to some magnesium alloys, and the corrosion resistance was better than that of extruded high-purity magnesium [55].

Due to the combined effect of grain refinement, texture and dislocation strengthening, the alloy obtained good mechanical properties, with yield strength of 250±22 MPa, elongation of 25.37±0.79%, and low corrosion rate of 0.004-0.01 mm/year [60].

**Question 4: The inert Ti and degradable Mg and Zn have quite different mechanical and corrosion properties. The discussion may be made more detailed based on the refined grain, intermetallic compounds, texture, and materials.**

*Authors added the data of grain size in table 1 as well as the following info in the second paragraph of Section 2.1.*

Previous study has found that CP-Ti rods treated by both rotary swaging and annealing achieved a strong <10-10> fiber texture, a microstructure characteristics of UFG, while obtaining excellent mechanical properties including tensile strength of 870 MPa, elongation of 8.5%, and high fatigue limit of 490 MPa, which are very close to those of the Ti-6Al-4V alloy [19].

*Authors also added following discussions on refined grain, intermetallic compounds and texture.*

*The first paragraph of Section 2.2*

Current research topics include the development of new magnesium and magnesium alloys with good corrosion resistance, improving the mechanical properties of pure magnesium through material processing technology, and reducing the corrosion of magnesium alloys through coating technology [36,37]. The corrosion of magnesium and magnesium alloys is affected by many factors, such as: composition, structures including grain size, second phase, texture, dislocation and twin, processing technologies such as extrusion, rolling, casting, forging and heat treatment, chemical, mechanical and biological environment [7]. In order to improve the mechanical properties of magnesium and its alloys, one of the solutions is to add alloying elements such as Zn, Ca, Sr, Li, and Nd [38], which can improve the effect of both solid solution and precipitation strengthening, but most of as-generated intermetallic compounds as the second phase will increase corrosion, due to the potential difference between the second phase and the α-Mg matrix. As a result, pitting corrosion, filament corrosion and exfoliation corrosion could accelerate the degradation of the alloy [39,40].

*The second paragraph of Section 2.2*

Fu et al. found that due to the strengthening effect brought by grain refinement, the mechanical properties and corrosion resistance of high-purity magnesium were also significantly improved, with the strength reaching 210MPa, which is comparable to some magnesium alloys, and the corrosion resistance was better than that of extruded high-purity magnesium [55]. This suggests great potentials for product design and performance improvement of pure magnesium. Meanwhile, new evaluation methods are constantly improving. For example, new testing technologies such as thin electrolyte layer model, to simulate the in vivo corrosion of pure magnesium and other degradable materials have been developed, which could provide a convenient and efficient method for future product development.

*The third paragraph of Section 2.2*

Due to the combined effect of grain refinement, texture and dislocation strengthening, the alloy obtained good mechanical properties, with yield strength of 250±22 MPa, elongation of 25.37±0.79%, and low corrosion rate of 0.004-0.01 mm/year [60].

*The second to the last paragraph of Section 2.2*

At the same time, with the application of novel processing technologies including ECAP, rotary swaging (RS) and thermo-mechanical treatment (TMT) in metallic biomaterials such as titanium, magnesium and zinc, and the research on the deformation mechanism, enhancement of mechanical property and improvement of corrosion resistance of pure metals or simple alloys, the effects of grain refinement, texture and wide stacking faults on materials’ performance have been thoroughly investigated and gradually revealed[55,56]. In addition, the application of RS may overcome the limitation of ECAP and HPT regarding the small size (especially length) of as-prepared materials, which could provide new opportunities for the promotion and application of PMBs [19].

**Question 5: It is better to give an illustration drawn by yourself, including the materials knowledge system.**

*Authors created the following graph of ultimate tensile strength-fatigue strength in Figure 2 based on the data collected in Table 1.*

**Question 6: 4. Conclusions. The review article may have a “Summary and future perspective”. This part needs a revision. It should be of scientific perspectives.**

*Please note this article is not a Review, but a Perspective. Because Perspective has its own format, which includes conclusions but not Summary and future perspective. In addition, contents in this manuscript such as Section 3.1 are the scientific perspectives that the reviewer is referring to. As a result, authors decided not to add “summary and future perspective”.*

**Question 7: Following important references should be cited:
[1] Biomedical Magnesium Alloys: Composition, Microstructure and Corrosion. Rong-Chang Zeng, Lan-Yue Cui, Wei Ke, Acta Metallurgica Sinica, 2018,54(9):1215-1235. In Chinese.
[2] Exfoliation corrosion. Zi-You Ding, et al. Journal of Materials Science and Technology, 34 (2018) 1550–1557.
[3] Corrosion types of magnesium alloys, Ed. Tomasz Tański, Magnesium Alloys. London, IntechOpen. 2018. DOI: 10.5772/intechopen.80083
[4] In vitro corrosion of pure magnesium and AZ91 alloy-The influence of thin electrolyte layer thickness. Regenerative Biomaterials, (2016)3 (1):49-56.[2]
[5] In vitro corrosion of as-extruded Mg-Ca alloys—The influence of Ca concentration. Corrosion Science, 2015, 96 (2015) 23–31.
[6] Corrosion and characterisation of dual phase Mg-Li-Ca alloy in Hank’s solution: the influence of microstructural features. Corrosion Science,79, 2014: 69-82.
[7] Advances in antibacterial coatings and magnesium alloys–A review. Acta Metallurgica Sinica (English Letters), 33, 2020, 615-629.
[8] Recent progress in layer-by-layer self-assembled coatings upon biodegradable magnesium alloys. Science China Materials, 64, (2021)2093–2106
[9] Dealloying corrosion of anodic and nanometric Mg41Nd5 in solid solution-treated Mg-3Nd-1Li-0.2Zn alloy. Journal of Materials Science & Technology, 83, 2021, 161-178.**

*The recommended references were ALL added according to the scientific content of this manuscript as following.*

7.	Zeng R, Cui L-Y, Ke W. Biomedical Magnesium Alloys: Composition, Microstructure and Corrosion. *Acta Metall Sin* 2018;54:1215-1235. In Chinese.

35.	Shao Y, Zeng R-C, Li S-Q *et al.* Advance in Antibacterial Magnesium Alloys and Surface Coatings on Magnesium Alloys: A Review. *Acta Metall Sin (English Letters)* 2020;33:615-629.

36.	Zeng R-C, Sun L, Zheng Y-F *et al.* Corrosion and characterisation of dual phase Mg–Li–Ca alloy in Hank’s solution: The influence of microstructural features. *Corros Sci* 2014;79:69-82.

37.	He L-j, Shao Y, Li S *et al.* Advances in layer-by-layer self-assembled coatings upon biodegradable magnesium alloys*. Sci China Mater* 2021;64:2093 - 2106.

38.	Gao G-J, Zeng M-Q, Zhang E-L *et al.* Dealloying corrosion of anodic and nanometric Mg41Nd5 in solid solution-treated Mg-3Nd-1Li-0.2Zn alloy. *J Mater Sci Technol* 2021;83:161-178.

39.	Zeng R-C, Qi W-C, Cui H-Z *et al.* In vitro corrosion of as-extruded Mg–Ca alloys—The influence of Ca concentration. *Corros Sci* 2015;96:23-31.

40.	Ding Z-Y, Cui L-Y, Zeng R-C *et al.* Exfoliation corrosion of extruded Mg-Li-Ca alloy. *J Mater Sci Technol* 2018;34:1550-1557.

41.	Rong-Chang Z, Zheng-Zheng Y, Xiao-Bo C *et al.* Corrosion Types of Magnesium Alloys. In: Tomasz T, Wojciech B, Mariusz K (eds). *Magnesium Alloys*. Rijeka: IntechOpen, 2018, Ch. 3.

56.	Zeng RC, Qi WC, Zhang F *et al.* In vitro corrosion of pure magnesium and AZ91 alloy-the influence of thin electrolyte layer thickness. *Regen Biomater* 2016;3:49-56.

**Reviewer #4:**

**The article tries to define a new concept, plain metallic biomaterials (PMBs), and makes explanations for it throughout the context, even giving two examples. However, this concept is not novel and innovative, and even is meaningless, based on the following analyses:
1. This concept learnt from a paper that proposed the concept of plain materials, and metallic biomaterials are just one class of materials belonging to it.**

*The reviewer is correct that the concept of PMB is derived from PM, which has already described in the Introduction. However, authors would like to point out that the concept of PMB is the development and expansion of that of PM. Meanwhile, for biomaterials and implantable devices, PMB is a new concept and an innovative research field with huge translation potential. As a result, authors believe that our manuscript is meaningful and novel.*

**2. Materials, especially metallic materials, with simple chemical compositions and satisfied properties have been one of important targets for material scientists to pursue, even for biomaterials. This was why pure titanium and magnesium with ultra-fined grains were developed, as mentioned in the article.**

*The reviewer is correct. The fast development of pure Ti and Mg with UFGs is exactly one of the reasons that authors propose the concept of PMBs.*

**3. However, if a metallic biomaterial with simple composition, for instance, pure titanium as mentioned in the article, needs a complex or costly processing for fabrication, such as the severe plastic deformation (SPD) technology described in the articles, it is also meaningless for practical application.**

*The reviewer is correct. New processing technologies and costs are representative challenges to achieve ideal PMBs. In fact, such challenges have been discussed in Section 3.2 of this manuscript. However, just because of these challenges, scientists and engineers are encouraged to develop better technologies and more applications so as to both create new opportunities and overcome challenges. One of the examples is additive manufacturing such as 3D printing. At its seminal stage, 3D printing has been questioned about its process efficiency and costs. With the development of technology and applications, 3D printing has now been widely used in the medical field.*

**4. As those two examples mentioned in the article are already existed metallic biomaterials without novelty, there is no need for change of current regulatory policy, as proposed in the article, unless the PMBs concept can promote the development of some new materials.**

*Although the two examples described in the manuscript are already existing PMBs, there is no medical product of these materials approved in both China and the US. As a result, it is necessary to conduct research and development of novel PMBs as well as research on regulatory science for medical devices to provide not only materials for new medical products, but also improved policies and approaches for regulatory approval.*

**5. What we expect for this article is to propose a novel idea to develop new material systems with unimaginable performance, instead of the currently existed materials.**

*Based on existing materials as representative examples, this manuscript proposes the concept of PMBs, which presents both challenges and opportunities, to encourage biomaterials scientists to develop novel materials and medical devices with better performance. Meanwhile, the opportunities and challenges were also discussed from the perspective of regulatory in this manuscript (section 3.3). The incentive policies and promotion measures of the regulatory authorities for new materials and devices will also be applied to the research and development of more novel material systems, so as to further promote the development of material processing technology and the emergence of new materials.*
